# Prognosis and risk factors for malignant peripheral nerve sheath tumor: a systematic review and meta-analysis

**DOI:** 10.1186/s12957-020-02036-x

**Published:** 2020-09-30

**Authors:** Zhenyu Cai, Xiaodong Tang, Haijie Liang, Rongli Yang, Taiqiang Yan, Wei Guo

**Affiliations:** Musculoskeletal Tumor Center, Peking University People’s Hospital, No. 11 Xizhimen South Street, Xicheng District, Beijing, 100044 China

**Keywords:** Malignant peripheral nerve sheath tumor, Prognosis, Local recurrence, Risk factors, Meta-analysis

## Abstract

**Background:**

No available meta-analysis was printed to systematically introduce the MPNST clinic outcome and risk factors based on largely pooled data. This systematic review and meta-analysis aimed to investigate 5-year OS rate, 5-year EFS rate, and LR rate for MPNST, and to assess potential risk factors for prognosis.

**Methods:**

Electronic articles published between January 1, 1966 and February 29, 2020 were searched and critically evaluated. The authors independently reviewed the abstracts and extracted data for 5-year OS rate, 5-year EFS rate, LR rate, and potential risk factors for prognosis.

**Results:**

Twenty-eight literatures were finally included for meta-analysis. The pooled 5-year OS rate, 5-year EFS rate, and LR rate were 49%, 37%, and 38%, respectively. The significant prognostic factors for survival were *NF1* status, tumor size, depth, location, malignant grade, margin status, chemotherapy, and radiotherapy. Age and sex were not associated with survival.

**Conclusion:**

Survival and local recurrence of MPNST are poor. Worse prognosis is mainly associated with *NF 1*, large size, deep to fascia, high grade, metastases, and location (trunk and head and neck). Complete resection with adequate surgical margins is the mainstay protective factor of MPNST patients, following necessary adjuvant therapies.

## Introduction

Malignant peripheral nerve sheath tumor (MPNST) is a rare malignant mesenchymal lesion that accounts for 5% to 10% of all soft tissue sarcoma [1, 2]. Further, 50–60% of patients with MPNST are associated to neurofibromatosis type 1 (*NF1*); others are radiation-induced or sporadic [3]. The behavior of MPNST is badly aggressive with high local recurrence rate and poor survival. Resection surgery is the main therapy for MPNST, while radiation and systemic chemotherapy was also widely used despite their uncertain effect. Radical therapy surgery combined with adjuvant therapy has been applied past decades. However, the prognosis for MPNST patients remains truly dissatisfactory, with 5-year overall survival (OS) rate of 15–66% [1, 4–10], 5-year event-free survival (EFS) of 24–53% [6, 11–17], and local recurrence (LR) rate of 20% to 85.7% [2, 4, 18–20]. It was obvious that the reported rates varied widely in different literatures.

As for genetic and pathology of MPNST, recurrent genetic mutations have been identified in recent studies, such as loss-of function in *NF1*, *PRC2*, *TP53*, *CDKN2A*, which may provide new opportunities for therapeutic intervention [21]. De Raedt et al. [22] revealed the loss of *SUZ12* strengthened effects of *NF1* mutations by amplifying Ras-driven transcription through effects on chromatin. Zhang et al. [23] also reported somatic mutations of *SUZ12* in MPNST. Lee et al. [24] reported that *PRC2* was recurrently inactivated through *EED* or *SUZ12* loss in MPNST. Sohier et al. [25] confirmed the frequent biallelic inactivation of *PRC2* subunits *SUZ12* and *EED* in MPNST, and suggests the implication of *KDM2B* in *NF1*-associated MPNST. Cleven et al. [26] revealed that loss of *H3K27* tri-methylation was related to poorer survival in MPNST.

Given the rarity of MPNST, there are sporadic published studies [14, 16, 27–29] reporting the prognosis and related factors. Although poor survival may result from large tumor size [13, 30, 31], inadequate margin [13, 15, 30], high-level tumor grade [13–15], or presence of distant metastasis [13, 31, 32], prognostic factors have not reached an agreement in literatures. Besides, few prognostic factors were reported in each study, and the population of these studies was not enough to indicate more detail factors. Above all, no available meta-analysis was printed to systematically introduce the MPNST clinic outcome and risk factors based on largely pooled data.

Therefore, we performed a systematic review and meta-analysis to provide the most up-to-date estimates of the 5-year OS rate, 5-year EFS rate, and LR rate for MPNST. The study also assessed potential risk factors for prognosis.

## Materials and methods

### Search strategy

This study was carried out after obtaining an approval from the institutional review board of our hospital. A comprehensive literature search was performed using the PubMed, EMBASE, Web of Science, and Cochrane Library databases for studies published between January 1, 1966 and February 29, 2020. The following MeSH terms and their combinations were searched: (neurofibrosarcoma/malignant peripheral nerve sheath tumor/MPNST) and (recurrent/recurrence/prognosis/risk/relapse). Two authors (ZYC and XDT) independently reviewed the titles and abstracts to screen and extract relevant articles.

### Selection criteria

The PICOS criteria for inclusion and exclusion were as follows:

P (participants): Studies of MPNST with more than 30 patients were included.

I and C (intervention and control): Studies in which MPNST patients received treatments were included. If some studies included partially duplicated patients, only the studies which used the large and advanced data were included.

O (outcome): Studies that included the 5-year OS rate, 5-year EFS rate, or LR rate with or without the following clinicopathologic were included: gender, age, tumor size, depth, location, tumor grade, NF1, surgical margin, chemotherapy, and radiotherapy. For risk factor analysis, only the studies reporting the above rates with hazard radio (HR) and 95% confidence interval (CI) were included. When a literature reported the results on different subpopulations, we regarded it as separate studies in the meta-analysis.

S (study type): Research articles published between 1 January 1966 and 29 February 2020 were included. All review papers, meta-analysis, and case reports were excluded.

### Quality assessments

The quality of each eligible study was rated independently by two reviewers (ZYC and HJL) using the modified Newcastle–Ottawa scale [33]. A score of 0–9 was assigned to each study.

### Data extraction

A data collection sheet was developed to record the level of evidence, study quality, available outcomes, and risk factors. Two investigators (ZYC and XDT) independently extracted data from these studies. For age and tumor size, only the studies that used measurement data were included. If variable was divided into dichotomous subgroups, the two subgroups data was included no matter what the cutoff value was. If variable was divided into polytomous rather than dichotomous subgroups, only the date of subgroups in both ends was included. When describing survival, some studies used cause-specific survival (CSS) or disease-specific survival (DSS) instead of OS. CSS and DSS belong to OS, so both were regarded as OS during data extraction. What is more, some studies described event free survival (EFS) with disease-free survival (DFS), tumor-free survival (TFS), progression-free survival (PFS), so the related data was also extracted.

### Statistical analysis

The analyses were performed using Stata 14.0 (StataCorp, College Station, TX, USA). We used a random-effects model to produce a pooled overall estimate for the 5-year OS rate, 5-year EFS rate, and LR rate. The HR was used to compare dichotomous variables. All results were reported with 95% CI. Statistical heterogeneity between studies was assessed using the Cochran’s *Q* test and quantified using the *I*^2^ statistic. If *p* ≤ 0.1 or *I*^2^ ≥ 50%, the heterogeneity was considered as existing and the random-effect model was used to merge HR. If *p* > 0.1 and *I*^2^ > 50%, the fixed-effect model was used to merge the HR values. Random-effect model was used to perform subgroup analysis. When HR > 1, the factors were accepted as risk factors resulting in poor prognosis. When HR > 1, the factors were accepted as protective factors resulting in good prognosis. If there was significant heterogeneity, an increased quantity of included studies was necessary.

### Sensitivity analysis and publication bias

Sensitivity analysis was handled to evaluate whether the results of meta-analysis changed after the removal of any one study. To assess the presence of publication bias, we used Egger’s test. A value of *p* < 0.05 was considered statistically significant publication bias.

## Results

**Fig. 1:**
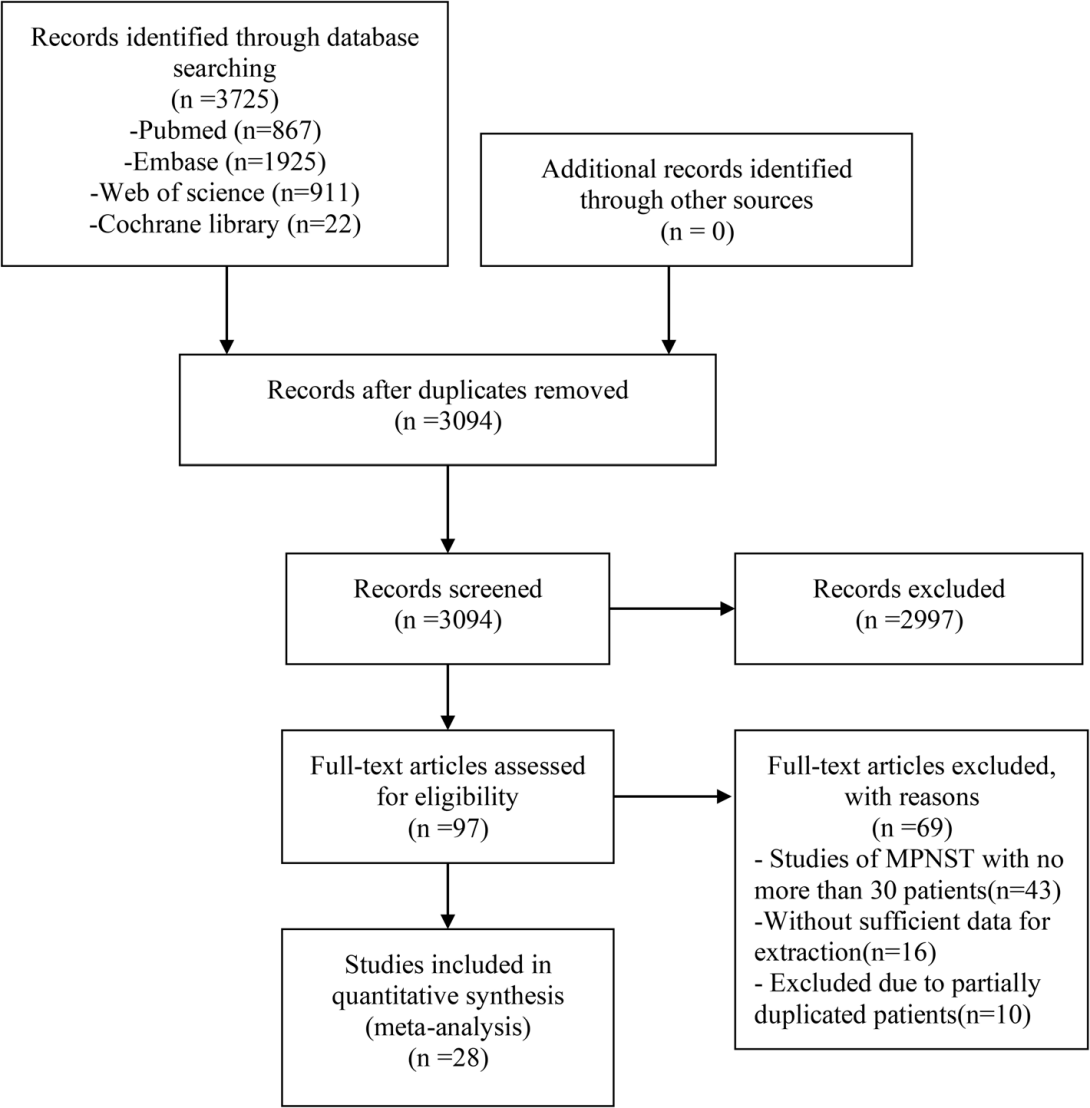
The flow chart showed the selection of studies for meta-analysis

We preliminarily screened 3725 literatures from PubMed, EMBASE, Web of Science, and Cochrane Library databases. After reading, 3697 literatures did not conform to inclusion criteria. Therefore, 28 literatures [6, 11–18, 27–32, 34–46] were finally included for meta-analysis (Fig. 1). All the included studies (Table 1) were retrospective and had an evidence of 3b or 4 according to the criteria of the Center for Evidence-Based Medicine in Oxford, UK [47]. All observation studies had a quality score of 5 or higher (Newcastle-Ottawa scale) and were considered to have high quality.

**Table 1:** Characteristics of the included studies

Study	Year	Time frame	Level of evidence^a^	Quality score^b^	Country	Age (years)^c^	Total pts. (*n*)	Male	Female	Median follow-up (months)	Outcome
Martin, E. et al.	2020	1989–2017	3b	7	Netherlands	49	784	421	363	NA	OS
Yan, P. H. et al.	2019	1973–2014	3b	6	China^d^	45	689	340	349	NA	OS
van Noesel, M. M. et al.	2019	2005–2016	3b	7	Five European countries	13.7	51	25	26	64.6	OS/EFS/LR
Shurell-Linehan, E. et al.	2019	1974–2012	4	7	USA	32.5	38	30	8	12.5	DSS/DFS
Mowery, A. et al.	2019	2004–2015	3b	6	USA^e^	47	2858	1554	1304	30.5	OS
Miao, R. Y. et al.	2019	1960–2016	3b	7	USA	41	280	138	142	43.1	OS/PFS/LR
Bergamaschi, L. et al.	2018	1979–2004	3b	7	Italy	< 21	73	37	36	NA	OS/LR
Yuan, Z. N. et al.	2017	1999–2016	3b	6	China	40	159	81	78	31	OS/TFS/LR
Watson, K. L. et al.	2017	1990–2014	3b	7	USA	37	289	155	134	25.56/ 26.16/ 20.88 ^f^	DSS /LR
Vasconcelos, R. A. T. et al.	2017	1990–2010	4	7	Brazil	43.5	92	41	51	24.8	OS/LR
Hwang, I. K. et al.	2017	1988–2015	3b	7	Korea	40.4	95	50	45	NA	OS
Valentin, T. et al.	2016	1990–2013	3b	7	France	42	340	190	150	87.6	OS/DFS
Wang, T. et al.	2015	2001–2012	4	7	China	50	43	25	18	24	OS/LR
Ma, C. et al.	2014	1996–2012	4	6	China	41	43	25	18	NA	OS/LR
Goertz, O. et al.	2014	1991-2004	4	5	Germany	54	65	32	33	36	OS /LR
Fan, Q. et al.	2014	NA	3b	6	China	40	146	79	67	NA	OS/TFS
LaFemina, J. et al.	2013	1982–2011	3b	6	USA	38	105	71	34	31.2	DSS/LR
Kamran, S. C. et al.	2013	1999–2011	3b	7	USA	43.2	84	47	37	19	LR
Stucky, C. C. et al.	2012	1985–2010	3b	7	USA	44	175	85	90	74	DSS/LR
Rekhi, B. et al.	2010	2002–2006	4	5	India	39	63	46	17	NA	LR
Longhi, A. et al.	2010	1969–2008	3b	7	Italy	39	62	39	23	54	OS/DFS/LR
Porter, D. E. et al.	2009	1979–2002	3b	7	UK	26/53^g^	123	NA	NA	6–252^h^	OS/LR
Keizman, D. et al.	2009	1994–2006	4	6	Israel	41	46	30	16	47	LR
Okada, K. et al.	2007	1994–2002	3b	7	Japan	45	56	22	34	41	OS/LR
Anghileri, M. et al.	2006	1976–2003	3b	7	Italy	37	205	108	97	NA	CSS/LR
Carli, M. et al.	2005	1975–1998	3b	6	Germany and Italy	11	167	83	84	87.6	OS/PFS
Meis, J. M. et al.	1992	1965–1985	4	7	USA	10	47	42	36	22	LR
Nambisan, R. N. et al.	1984	1971–1981	4	5	USA	35	31	16	15	NA	LR

*NA* not available, *OS* overall survival, *CSS* cause-specific survival, *DSS* disease-specific survival, *EFS* event-free survival, *DFS* disease-free survival, *TFS* tumor-free survival, *PFS* progression-free survival, *LR* local recurrence

^a^Level of evidence: according to the criteria of the Centre for Evidence-Based Medicine

^b^Quality score: the score of the study using the Newcastle–Ottawa Scale

^c^Age is represented by the median, or the average, or the range age of the study population

^d^Yan, P. H. et al. (2019) used the data of SEER database

^e^Mowery, A. et al. (2019) used the data of NCDB

^f^The median follow-up interval was 25.56 for sporadic, 26.16 for NF1-associated, and 20.88 for RT-associated MPNST

^g^The median age was 26 for NF1 MPNST, 53 for sporadic MPNST

^h^The follow-up time ranged from 6 to 252 months

### 5-year OS rate, 5-year EFS rate, and LR rate

The pooled data of 5-year OS rate consist of 22 studies [6, 11–18, 27, 28, 30–32, 34–38, 40, 42, 44] with 6742 patients. The 5-year OS rate was 49% (95%CI 45–53%). The pooled data of 5-year EFS rate consist of 8 studies [6, 11–17] with 1243 patients. The 5-year EFS rate was 37% (95%CI 32–43%). The pooled data of LR rate consist of 19 studies [11, 13, 14, 17, 27–29, 34–36, 38–46] with 1738 patients. The LR rate was 38% (95%CI 30–47%) (Table 2; Figure 1S).

**Table 2:** 5-year OS rate, 5-year EFS rate, and LR rate of MPNST

Indicators	N	N of pts.	Rate range	ES	95% CI	Heterogeneity (*I*^2^)	Model	*p*	Sensitivity analysis	Affected study	Publication bias (Egger’s test)
5-year OS rate [6, 11–18, 27, 28, 30–32, 34–38, 40, 42, 44]	22	6742	16–63%	49%	45–53%	85.3%	Random	0.000	No effect	None	0.279
5-year EFS rate [6, 11–17]	8	1243	24–53%	37%	32–43%	73.2%	Random	0.000	No effect	None	0.516
LR rate [11, 13, 14, 17, 27–29, 34–36, 38–46]	19	1738	13%-86%	38%	30%-47%	92.9%	Random	0.000	No effect	None	0.748

### Prognostic factors

The prognostic factors with similar variables were pooled in the meta-analysis. The details of meta-analysis results are shown in Table 3 and Figure 2S.

**Table 3:** Show results of meta-analysis including pooled HR, 95% CI, sensitivity analysis, and publication bias

Prognostic factors	*N*	HR range	Pooled HR	Pooled 95% CI	Heterogeneity (*I*^*2*^)	Model	*p*	Sensitivity analysis	Affected study	Publication bias (Egger’s test)
The male vs. the female [16, 29–31, 35]	7	0.65–3.87	1.09	0.87–1.37	55.3%	Random	0.466	No effect	None	0.556
The older vs. the younger [14, 16, 29–32, 35, 37]	9	0.63–8.13	1.45	0.98–2.16	80.3%	Random	0.065	Effect	Yuan, Z. N. et al. [14], Hwang, I. K. et al. [37], Fan, Q. et al. [16], LaFemina, J. et al. [29]	0.284
NF 1 vs. non-NF 1 MPNST [11, 13–16, 18, 30, 34–37, 40, 42]	13	1.00–3.54	1.56	1.35–1.79	34.1%	Fixed	0.000	No effect	None	0.569
Large size vs. small size [13, 14, 17, 28, 30, 31, 35–37, 44]	10	1.04–7.88	1.93	1.62–2.29	44.4%	Random	0.000	No effect	None	0.059
Deep vs. superficial to fascia [14, 15, 24, 29, 34]	5	1.79–3.16	2.09	1.52–2.89	0.0%	Fixed	0.000	No effect	None	0.227
Trunk vs. extremity [15, 17, 18, 24, 34]	5	1.01–3.70	1.79	1.07–3.00	74.9%	Random	0.025	Effect	Stucky, C. C. et al. [40], Longhi, A. et al. [17], Anghileri, M. et al. [18]	0.041
Head and neck vs. extremity [14–16, 18, 30, 40]	6	1.10–4.15	1.38	1.07–1.78	30.3%	Fixed	0.014	Effect	Valentin, T. et al. [15]	0.047
Grade II vs. Grade I [13–16, 31]	6	0.89–2.99	1.43	1.06–1.91	43.7%	Fixed	0.017	Effect	Mowery, A. et al. [31], Miao, R. Y. et al. [13]	0.126
Grade III vs. Grade I [13–16, 31]	6	0.89–35.88	3.21	1.36–7.54	86.9%	Random	0.008	No effect	None	0.006
With vs. without metastases [13, 16, 31, 32, 44]	6	1.11–4.80	2.30	1.50–3.51	62.3%	Random	0.000	No effect	None	0.359
R1 vs. R0 resection [13, 15, 28, 30, 37]	6	0.98–5.93	1.31	1.04–1.64	22.3%	Fixed	0.022	Effect	Ma, C. et al. [28]	0.149
R2 vs. R0 resection [13–15, 18, 28–30, 35, 37]	10	0.97–13.15	2.40	1.96–2.95	0.0%	Fixed	0.000	No effect	None	0.019
With vs. without chemotherapy [14, 16, 18, 30, 32, 35, 40]	7	0.38–0.99	0.70	0.59–0.83	6.3%	Fixed	0.000	No effect	None	0.877
With vs. without radiotherapy [14–16, 18, 28, 30, 35]	7	0.31–0.96	0.65	0.49–0.88	50.0%	Random	0.005	No effect	None	0.006

### Sex

Seven studies (including subgroups) [16, 29–31, 35] comparing the overall survival between male and female were included. Values of *I*^2^ = 55.3% and *p =* 0.037 were obtained after the HR values of OS were merged, indicating that heterogeneity existed. A random-effect model was used to merge the HR = 1.09, 95% CI 0.87–1.37 and *p =* 0.466, suggesting that OS is of no significant difference in sex.

### Age

Nine studies (including subgroups) [14, 16, 29–32, 35, 37] compared the overall survival between the older and the younger subgroups. Values of *I*^2^ = 80.3% and *p =* 0.000 were obtained after the HR values of OS were merged, indicating that heterogeneity existed. A random-effect model was used to merge the HR = 1.45, 95% CI 0.98–2.16 and *p =* 0.065, showing no significant difference in the overall survival between the older and the younger subgroups.

### *NF 1* status

A total of 13 studies [11, 13–16, 18, 30, 34–37, 40, 42] assessed the association between *NF 1* and OS. Values of *I*^2^ = 34.1% and *p =* 0.110 were obtained after the HR values of OS were merged, indicating that heterogeneity did not exist. The pooled result via a fixed-effect model indicated that patients with *NF 1*-associated MPNST have poorer survival than patients with non-*NF 1*-associated MPNST (HR 1.56, 95%CI 1.35–1.79, *p =* 0.000).

### Tumor size

For tumor size, the terms “large” and “small” are relative concept. For example, if the tumor size was divided into two sub-groups named “< 5 cm” and “≥ 5 cm,” the “large” represented “≥ 5 cm” and the “small” represented “< 5 cm.” Ten studies [13, 14, 17, 28, 30, 31, 35–37, 44] evaluated tumor size (the large vs. the small) as a risk factor for OS. Values of *I*^2^ = 44.4% and *p =* 0.063 were obtained after the HR values of OS were merged, indicating that heterogeneity existed. A random-effect model was used to merge the HR = 2.08, 95% CI 1.59–2.71 and *p =* 0.000. The results showed that large tumor size correlated with a significantly higher risk for poor prognosis.

### Tumor depth

Five studies [14, 15, 30, 35, 40] evaluated relation between tumor depth related to fascia and OS. Values of *I*^2*-*^= 0.00% and *p =* 0.744 were obtained after the HR values of OS were merged, indicating that heterogeneity did not exist and a fixed-effect model was applied. Collectively, deep to fascia versus superficial to fascia significantly increased the risk of poor prognosis (HR 2.09, 95% CI 1.52–2.89, *p =* 0.000).

### Tumor site

A total of 6 studies [14–16, 18, 30, 40] evaluated the relation between the tumor site and OS. Five studies [15, 17, 18, 30, 40] compared the OS between trunk and extremity, with heterogeneity existing (*I*^2^ = 74.9% and *p =* 0.003) and a random-effect model applied. Trunk versus extremity had an increasing risk of bad prognosis (HR 1.79, 95% CI 1.07–3.00, *p =* 0.025). Six studies [14–16, 18, 30, 40] compared the OS between head and neck and extremity, with no heterogeneity existing (*I*^2^ = 30.3% and *p =* 0.208) and a fixed-effect model applied. Head and neck versus extremity had an increasing risk of bad prognosis (HR 1.38, 95% CI 1.07–1.78, *p =* 0.014).

### Tumor grade

A total of 6 studies (including subgroups) [13–16, 31] assessed the association between the tumor grade and OS. Most of the studies [14, 16, 31] used AJCC 8 Stage (The American Joint Committee on Cancer released updated cancer staging in 2017—known as AJCC 8) to define tumor grade. Six studies (including subgroups) [13–16, 31] compared the OS between grade II and I, without heterogeneity existing (*I*^2^ = 43.7% and *p =* 0.114) and a fixed-effect model applied. Grade II had an increasing risk of poor prognosis compared to grade I (HR 1.43, 95% CI 1.06–1.91, *p =* 0.017). Six studies (including subgroups) [13–16, 31] compared the OS between grade III and I, with heterogeneity existing (*I*^2^ = 86.9% and *p =* 0.000) and a random-effect model applied. Grade III had an increasing risk of poor prognosis compared to grade I (HR 3.21, 95% CI 1.36–7.54, *p =* 0.008).

### Metastases

There were 6 studies (including subgroups) [13, 16, 31, 32, 44] exploring OS and metastases included, with heterogeneity existing (*I*^2^ = 62.3% and *p =* 0.021) and a random-effect model applied. Patients with metastasis had poorer OS than those without (HR 2.30, 95% CI 1.50–3.51, *p =* 0.000).

### Surgical margin

A total of 10 studies (including subgroups) [13–15, 18, 28–30, 35, 37] estimated surgical margin as a factor influencing OS. Most studies used R0, R1, and R2 to describe margin status, while some used negative and positive. When performing meta-analysis, we regard negative and positive as R0 and R2, respectively. Six studies (including subgroups) [13, 15, 28, 30, 37] compared the OS between R1 and R0, with no heterogeneity existing (*I*^2^ = 22.3% and *p =* 0.267) and a fixed-effect model applied. R1 had an increasing risk of poor prognosis compared to R0 (HR 1.31, 95%CI 1.04–1.64, *p =* 0.022). Ten studies (including subgroups) [13–15, 18, 28–30, 35, 37] compared the OS between R2 and R0, with no heterogeneity existing (*I*^2^ = 0.0% and *p =* 0.511) and a fixed-effect model applied. R2 had an increasing risk of poor prognosis compared to R0 (HR 2.40, 95% CI 1.96–2.95, *p =* 0.000).

### Chemotherapy

Seven studies [14, 16, 18, 30, 32, 35, 40] evaluated relation between chemotherapy and OS. Values of *I*^2^ = 6.3% and *p =* 0.379 were obtained after the HR values of OS were merged, indicating that heterogeneity did not exist and a fixed-effect model was applied. Collectively, chemotherapy for MPNST is a significantly protective factor for OS (HR 0.70, 95% CI 0.59–0.83, *p =* 0.000).

### Radiotherapy

Seven studies [14–16, 18, 28, 30, 35] evaluated relation between chemotherapy and OS. Values of *I*^2^ = 50.0% and *p =* 0.062 were obtained after the HR values of OS were merged, indicating that heterogeneity existed and a random-effect model was applied. Collectively, radiotherapy for MPNST is a significantly protective factor for OS (HR 0.65, 95% CI 0.49–0.88, *p =* 0.005).

To test the influence of period on heterogeneity, the subgroup analyses were carried out to investigate the sources of heterogeneity (Tables 4 and 5).

**Table 4:** Subgroup analysis of 5-year OS rate, 5-year EFS rate, and LR rate of MPNST

Indicators	Subgroup (by study year)	Rate range	ES	95% CI	Heterogeneity (*I*^2^)	Model	*p*
5-year OS rate [6, 11–18, 27, 28, 30–32, 34–38, 40, 42, 44]		16–63%	49%	45–53%	85.3%	Random	0.000
	2016–2020	16–63%	49%	44–55%	90.6%	Random	0.000
	2005–2015	38–60%	49%	44–54%	63.4%	Random	0.000
5-year EFS rate [6, 11–17]		24–53%	37%	32–43%	73.2%	Random	0.000
	2016–2019	34–53%	41%	35–46%	54.5%	Random	0.000
	2005–2014	24–37%	30%	22–39%	68.7%	Random	0.000
LR rate [11, 13, 14, 17, 27–29, 34–36, 38–46]		13%-86%	38%	30%-47%	92.9%	Random	0.000
	2017–2019	18–86%	42%	25–60%	96.7%	Random	0.000
	1984–2015	13–60%	36%	29–43%	92.9%	Random	0.000

**Table 5:** Show prognostic factors results of subgroup meta-analysis of pooled HR, 95% CI

Prognostic factors	Subgroups (by study year)	*N*	HR range	Pooled HR	Pooled 95% CI	Heterogeneity (*I*^2^)	Model	*p*
The male vs. the female [16, 29–31, 35]		7	0.65–3.87	1.09	0.87–1.37	55.3%	Random	0.466
	2017–2020	5	0.65–3.87	1.01	0.75–1.36	62.4%	Random	0.956
	2013–2014	2	1.23–1.62	1.28	0.98–1.68	0.0%	Random	0.069
The older vs. the younger [14, 16, 29–32, 35, 37]		9	0.63–8.13	1.45	0.98–2.16	80.3%	Random	0.065
	2017–2020	7	0.81–8.13	1.67	1.10–2.54	81.0%	Random	0.017
	2013–2014	2	0.63–0.83	0.79	0.47–1.35	0.0%	Random	0.391
NF 1 vs. non–NF 1 MPNST [11, 13–16, 18, 30, 34–37, 40, 42]		13	1.00–3.54	1.56	1.35–1.79	34.1%	Fixed	0.000
	2016–2020	9	1.00–3.54	1.56	1.20–2.02	52.9%	Random	0.001
	2006–2014	4	1.37–1.96	1.68	1.26–2.25	0.0%	Random	0.000
Large size vs. small size [13, 14, 17, 28, 30, 31, 35–37, 44]		10	1.04–7.88	1.93	1.62–2.29	44.4%	Random	0.000
	2017–2020	7	1.04–2.99	1.74	1.45–2.10	0.0%	Random	0.000
	2007–2014	3	4.20–7.88	5.18	2.90–9.24	0.0%	Random	0.000
Deep vs. superficial to fascia [14, 15, 24, 29, 34]		5	1.79–3.16	2.09	1.52–2.89	0.0%	Fixed	0.000
	2016–2020	4	1.79–3.16	2.09	1.51–2.90	0.0%	Random	0.000
	2012	1	2.10	2.10	0.27–16.22	–	–	0.477
Trunk vs. extremity [15, 17, 18, 24, 34]		5	1.01–3.70	1.79	1.07–3.00	74.9%	Random	0.025
	2016–2020	2	1.01–1.26	1.05	0.83–1.32	0.0%	Random	0.684
	2006–2012	3	2.54–3.70	2.75	1.79–4.21	0.0%	Random	0.000
Head and neck vs. extremity [14–16, 18, 30, 40]		6	1.10–4.15	1.38	1.07–1.78	30.3%	Fixed	0.014
	2016–2020	3	1.11–3.25	1.47	0.88–2.47	41.4%	Random	0.145
	2006–2014	3	1.10–4.15	1.81	0.98–3.34	21.0%	Random	0.057
Grade II vs. Grade I [13–16, 31]		6	0.89–2.99	1.43	1.06–1.91	43.7%	Fixed	0.017
	2016–2019	5	0.89–2.99	1.63	0.96–2.77	52.8%	Random	0.071
	2014	1	1.85	1.85	0.80–4.26	–	–	0.151
Grade III vs. Grade I [13–16, 31]		6	0.89–35.88	3.21	1.36–7.54	86.9%	Random	0.008
	2016–2019	5	0.89–35.88	3.30	1.20–9.05	88.1%	Random	0.021
	2014	1	3.14	3.14	1.45–6.83	–	–	0.004
With vs. without metastases [13, 16, 31, 32, 44]		6	1.11–4.80	2.30	1.50–3.51	62.3%	Random	0.000
	2019	4	1.11–3.65	2.39	1.46–3.89	63.5%	Random	0.000
	2007–2014	2	1.50–4.80	2.34	0.77–7.10	63.7%	Random	0.134
R1 vs. R0 resection [13, 15, 28, 30, 37]		6	0.98–5.93	1.31	1.04–1.64	22.3%	Fixed	0.022
	2016–2020	5	0.98–2.27	1.25	0.99–1.58	0.0%	Random	0.058
	2014	1	5.93	5.93	1.50–23.49	–	–	0.011
R2 vs. R0 resection [13–15, 18, 28–30, 35, 37]		10	0.97–13.15	2.40	1.96–2.95	0.0%	Fixed	0.000
	2016–2020	7	1.89–7.63	2.34	1.84–2.97	0.0%	Random	0.000
	2006–2014	3	1.86-13.15	3.15	1.46–6.92	64.3%	Random	0.004
With vs. without chemotherapy [14, 16, 18, 30, 32, 35, 40]		7	0.38–0.99	0.70	0.59–0.83	6.3%	Fixed	0.000
	2017–2020	4	0.63–0.99	0.75	0.60–0.94	28.0%	Random	0.013
	2006–2014	3	0.38–0.70	0.56	0.37–0.86	0.56	Random	0.008
With vs. without radiotherapy [14–16, 18, 28, 30, 35]		7	0.31–0.96	0.65	0.49–0.88	50.0%	Random	0.005
	2016–2020	4	0.64–0.96	0.87	0.72–1.05	0.0%	Random	0.136
	2006–2014	3	0.31–0.53	0.45	0.30–0.67	0.0%	Random	0.000

Because there were rare studies discussing the risk factors of EFS and LR, so the meta-analysis associated with risk factors of EFS and LR was not performed.

### Sensitivity analysis and publication bias

The sensitivity analysis was performed in these groups. The pooled HR of age became statistical significance when to exclude one of these studies including Yuan Z. N. et al. [14], Hwang I. K. et al. [37], Fan Q. et al. [16], and LaFemina J. et al. [29]. The pooled HR of tumor site (Trunk vs. Extremity) became no statistical significance when to exclude one of these studies including Stucky C. C. et al. [40], Longhi A. et al. [17], and Anghileri M. et al .[18]. The pooled HR of tumor site (head and neck vs. extremity) became no statistical significance when to exclude Valentin T. et al. [15]. The pooled HR of tumor grade (grade II vs. I) became no statistical significance when to exclude one of Mowery A. et al. [31] and Miao R. Y. et al. [13]. The pooled HR of surgical margin (R1 vs. R0 resection) became no statistical significance when to exclude Ma, C. et al .[28]. The results of the other meta-analysis did not change after removal of any one research (Figure 3S and Figure 4S).

The Egger’s test was completed to examine the existence of publication bias. The possibilities of publication bias were found in tumor site (trunk vs. extremity, *p* = 0.041; head and neck vs. extremity, *p* = 0.047), tumor grade (grade III vs. I, *p* = 0.006), surgical margin (R2 vs. R0 resection, *p* = 0.019), and radiotherapy (with vs. without radiotherapy, *p =* 0.006). The Egger’s test resulted in *p* ≥ 0.05 in the other groups and indicated that the possibilities of publication bias can be excluded (Figure 5S and Figure 6S).

## Discussion

In 2002, WHO classified the original neurosarcoma, neurofibrosarcoma, and malignant Schwann cell tumor as MPNST. In 2013, MPNST was classified as soft tissue tumor, including two special subtypes—epithelioid malignant peripheral schwannoma and malignant Triton tumor (MTT). In recent years, increasing studies [11–13, 30–32, 48] had been performed to report the prognosis of MPNST and to explore the related factors of survival. However, the survival and recurrence of these patients were various in different literatures. Indeed, some factors were found as risk indicators in MPNST related literatures, such as *NF1* status [11, 13, 30, 48], negative margin [13, 30], and non-adoption of adjuvant therapy [28, 32], while several literatures [7, 14, 35, 49] failed to find prognostic power. Considering the disagreement on prognosis and risk factors, we performed the systematic review and meta-analysis to evaluate 5-year OS rate, 5-year EFS, and LR rate of MPNST, and to investigate the related risk factors for survival.

### 5-year OS rate, 5-year EFS, and LR rate

Although the prognosis of patients with MPNST is currently reported to be various in numerous studies, the 5-year survival of patients remains poor in general. Actually, 5-year OS rate was more than 50% in studies [15, 30–32] in which the participants were more than 300. For an Italian study focusing on pediatric patients (*n* = 73) with relapsing MPNST, Bergamaschi L. et al. [34] reported that the survival rate was 15.8% at 5 years which was the lowest rate in our included studies. For non-*NF1* MPNST children (*n* = 44) in the Netherlands, Martin E. et al. [48] showed the highest 5-year survival rate (75.8%) in these cohorts. In this study, the 5-year OS rate was 49% (range, 16–63%), which consisted with the most published studies.

The 5-year EFS rate was 34–40.6% in researches [6, 13–15] of which the number of patients exceed 150. For Chinese MPNST patients (*n* = 146), Fan Q. et al. [16] indicated that the tumor-free survival was 24% at 5 years with the median TFS time 25.64 months. This poor prognosis may be contributed to 41% of patients received subtotal resection rather than wide resection, *NF1* status, or large tumor size. In 2005, the European Pediatric Soft Tissue Sarcoma Group (EpSSG) developed a protocol specifically dedicated to nonrhab-domyosarcoma soft tissue sarcoma (NRSTS). The EpSSG-NRSTS-2005 study was a prospective European observational study for localized NRSTS for patients < 21 years of age, including patients with MPNST. For the study of MPNST based on EpSSG-NRSTS-2005, van Noesel M. M. et al. [11] included 51 patients with MPNST and reported 52.90% of 5-year EFS rate. This study reported the similar 5-year EFS rate (37%, range 24–53%) with other existing literatures.

Among sarcomas, MPNST has the highest recurrence rate [50]. Bergamaschi L. et al. [34] reported the highest LR rate of 86.3% in 73 patients, with 64.4% only local recurrence and 21.9% local and metastatic recurrence of MPNST. Keizman D. et al. [43] investigated 46 patients with MPNST, indicating 13% of patients occurred local recurrence with follow-up ranging from 3 to 120 months. This low LR rate may be attributed to the inadequate follow-up time and small population. In our study, although we found that the recurrence rates were currently reported to be different in numerous studies, the pooled LR rate was 37% (range, 13–86%). This result also revealed that aggressively effective therapy was necessary to be applied in order to control local recurrence.

### Risk factors for survival

The role of NF 1 status as a prognostic factor for MPNST remains highly debated. Kolberg M et al. [51] performed a meta-analysis in 2013 and indicated that NF 1 status had no effect on survival. Some studies [7, 18, 34, 37, 40] just identified a trend and reported the negative results based on large populations. With the improvement of survival for the NF1 patients in past years, they considered that the survival difference was diminishing. However, NF 1 status has been reported to be a risk factor for the prognosis of MPNST in some literatures [11, 30, 48]. Porter D. E. et al. [42] founded that NF 1 was independent predictor of poor outcome due to the genetic profile affecting aggressive potential, and emphasized the importance of NF 1 in MPNST staging. In our study, we confirmed that NF 1 status was the risk factor for the survival of MPNST.

Tumor size [13, 30, 44] and location [17, 18, 52, 53] have repeatedly been reported to affect survival, whereas tumor depth has only been shown an independent predictor of survival in two studies [15, 30]. The pooled result also proved that patients with larger tumor size has poorer prognosis than patients with smaller size. In our study, we found that compared with extremities site, both truncal site and head and neck locations were independently related to worse survival. Although some literature s[14, 35, 40] failed to show significantly statistic relationship between depth and prognosis, pooled data in this study showed that deep to fascia had poorer prognosis than superficial to fascia, which may be associated with more complex anatomical characteristics, larger extent of tumor, or intraoperative more massive bleeding.

The oncologic factors, including grade and metastases, were also evaluated in this study. As for prognosis, grade II or III were definitely worse than grade I, despite existing heterogeneity in meta-analysis. This may be attributed to few studies [13–16, 31] included, small population of research, and non-adoption of unitary classification like AJCC stage. Besides, we showed that patients with metastases had poorer prognosis than those without metastases via the pooled data.

The main treatment for MPNST is surgical therapy [1, 18, 29], while the effect of adjuvant therapies on prognosis remains unclear [7, 40, 52]. Adequate margin is vital to control local recurrence, and then the survival of patients. Our study revealed that resection with negative margin was a protective factor for better prognosis, which is in accordance with previous literatures [13, 15, 18, 30, 37]. Besides, the pooled results also indicated chemotherapy and radiotherapy were important to improve survival. Therefore, adjuvant therapies are recommended for patients with MPNST, especially for patients with positive margins.

This meta-analysis had some limitations. First, our meta-analysis was based on retrospective studies, so selection bias cannot be avoided. Second, some studies with small sample were applied into prognostic factors analysis, which may lead to publication bias and affect sensitivity. Further studies may be needed to verify our conclusions. Furthermore, the follow-up time was various in each study. Besides, to review as many articles as possible, the period of publications was set as long as possible, which may result in heterogeneity. However, after reviewing those articles via selection criteria, most articles included in the study were published between 2005 and 2020. To dispel worries about the influence of period, we performed subgroup analysis. In spite of these limitations, this study applied a series of measures and strict standard to evaluate the quality of these studies.

## Conclusion

In conclusion, our results indicate that the survival and local recurrence of MPNST are poor. Worse prognosis is mainly associated with NF 1 mutation, large size, deep to fascia, high grade, metastases, and location (trunk and head and neck). Complete resection with adequate surgical margins is the mainstay protective factor of MPNST patients, following necessary adjuvant therapies.

## Supplementary information

**Additional file 1: Figure S1.** Forest plot showing the pooled rates: (A) 5-year OS rate. (B) 5-year EFS rate. (C) LR rate

**Additional file 2: Figure S2.** Forest plot showing the pooled HR of OS by prognostic factors: (A) Sex (The female vs. The male). (B) Age (The older *vs.* The younger). (C) NF 1 status (NF 1 *vs.* Non-NF 1 MPNST). (D) Tumor size (Large size vs. Small size). (E) Tumor depth (Deep *vs.* Superficial to fascia). (F) Tumor site (Trunk *vs.* Extremity). (G) (Head & neck *vs.* Extremity). (H) Tumor grade (Grade II *vs.* Grade I). (I) Tumor grade (Grade III *vs.* Grade I). (J) Metastases (With *vs.* Without). (K) Margin status (R1 *vs.* R0 resection). (L) Margin status (R2 *vs.* R0 resection). (M) Chemotherapy (With *vs.* Without). (N) Radiotherapy (With *vs.* Without)

**Additional file 3: Figure S3.** Forest plot for the sensitivity analysis in the meta-analysis: (A) 5-year OS rate. (B) 5-year EFS rate. (C) LR rate

**Additional file 4: Figure S4.** Forest plot for the sensitivity analysis in the meta-analysis: (A) Sex (The female *vs.* The male). (B) Age (The older *vs.* The younger). (C) NF 1 status (NF 1 *vs.* Non-NF 1 MPNST). (D) Tumor size (Large size *vs.* Small size). (E) Tumor depth (Deep *vs.* Superficial to fascia). (F) Tumor site (Trunk *vs.* Extremity). (G) (Head & neck *vs.* Extremity). (H) Tumor grade (Grade II *vs.* Grade I). (I) Tumor grade (Grade III *vs.* Grade I). (J) Metastases (With *vs.* Without). (K) Margin status (R1 *vs.* R0 resection). (L) Margin status (R2 *vs.* R0 resection). (M) Chemotherapy (With *vs.* Without). (N) Radiotherapy (With *vs.* Without)

**Additional file 5: Figure S5.** Egger’s test for publication bias in the meta-analysis: (a) 5-year OS rate. (b) 5-year EFS rate. (c) LR rate

**Additional file 6: Figure S6.** Egger’s test for publication bias in the meta-analysis: (A) Sex (The female *vs.* The male). (B) Age (The older *vs.* The younger). (C) NF 1 status (NF 1 *vs.* Non-NF 1 MPNST). (D) Tumor size (Large size *vs.* Small size). (E) Tumor depth (Deep *vs.* Superficial to fascia). (F) Tumor site (Trunk *vs.* Extremity). (G) (Head & neck *vs.* Extremity). (H) Tumor grade (Grade II *vs.* Grade I). (I) Tumor grade (Grade III *vs.* Grade I). (J) Metastases (With *vs.* Without). (K) Margin status (R1 *vs.* R0 resection). (L) Margin status (R2 *vs.* R0 resection). (M) Chemotherapy (With *vs.* Without). (N) Radiotherapy (With *vs.* Without)

## Data Availability

Please contact author for data requests. The manuscript submitted does not contain information about medical device(s)/drug(s).

## Abbreviations

MPNST: Malignant peripheral nerve sheath tumor
NF1: Neurofibromatosis type 1
OS: Overall survival
CSS: Cause-specific survival
DSS: Disease-specific survival
EFS: Event-free survival
DFS: Disease-free survival
TFS: Tumor-free survival
PFS: Progression-free survival
LR: Local recurrence

## References

[1] Ducatman BS, Scheithauer BW, Piepgras DG (1986). Malignant peripheral nerve sheath tumors. A clinicopathologic study of 120 cases. Cancer.

[2] Vauthey JN, Woodruff JM, Brennan MF (1995). Extremity malignant peripheral-nerve sheath tumors (neurogenic sarcomas)—a 10-year experience. Ann Surg Oncol.

[3] Wanebo JE, Malik JM, VandenBerg SR, Wanebo HJ, Driesen N, Persing JA (1993). Malignant peripheral nerve sheath tumors. A clinicopathologic study of 28 cases. Cancer.

[4] Zhu B, Liu X, Liu Z, Yang S, Liao HI, Jiang L, Wei F (2012). Malignant peripheral nerve sheath tumours of the spine: clinical manifestations, classification, treatment, and prognostic factors. Eur Spine J.

[5] Bishop AJ, Zagars GK, Torres KE, Bird JE, Feig BW, Guadagnolo BA (2018). Malignant peripheral nerve sheath tumors: a single institution's experience using combined surgery and radiation therapy. Am J Clin Oncol.

[6] Carli M, Ferrari A, Mattke A, Zanetti I, Casanova M, Bisogno G, Cecchetto G, Alaggio R, De Sio L, Koscielniak E (2005). Pediatric malignant peripheral nerve sheath tumor: the Italian and German soft tissue sarcoma cooperative group. J Clin Oncol.

[7] Zou CY, Smith KD, Liu J, Lahat G, Myers S, Wang WL, Zhang W, McCutcheon IE, Slopis JM, Lazar AJ (2009). Clinical, pathological, and molecular variables predictive of malignant peripheral nerve sheath tumor outcome. Ann Surg.

[8] Kahn J, Gillespie A, Tsokos M, Ondos J, Dombi E, Camphausen K, Widemann BC, Kaushal A (2014). Radiation therapy in management of sporadic and neurofibromatosis type 1-associated malignant peripheral nerve sheath tumors. Front Oncol.

[9] Wong WW, Hirose T, Scheithauer BW, Schild SE, Gunderson LL (1998). Malignant peripheral nerve sheath tumor: analysis of treatment outcome. Int J Radiat Oncol Biol Phys.

[10] Farid M, Demicco EG, Garcia R, Ahn L, Merola PR, Cioffi A, Maki RG (2014). Malignant peripheral nerve sheath tumors. Oncologist.

[11] van Noesel MM, Orbach D, Brennan B, Kelsey A, Zanetti I, de Salvo GL, Gaze MN, Craigie RJ, McHugh K, Francotte N (2019). Outcome and prognostic factors in pediatric malignant peripheral nerve sheath tumors: an analysis of the European Pediatric Soft Tissue Sarcoma Group (EpSSG) NRSTS-2005 prospective study. Pediatr Blood Cancer.

[12] Shurell-Linehan E, DiPardo BJ, Elliott IA, Graham DS, Eckardt MA, Dry SM, Nelson SD, Singh AS, Kalbasi A, Federman N (2019). Pathologic response to neoadjuvant therapy is associated with improved long-term survival in high-risk primary localized malignant peripheral nerve sheath tumors. Am J Clin Oncol.

[13] Miao RY, Wang HT, Jacobson A, Lietz AP, Choy E, Raskin KA, Schwab JH, Deshpande V, Nielsen GP, DeLaney TF (2019). Radiation-induced and neurofibromatosis-associated malignant peripheral nerve sheath tumors (MPNST) have worse outcomes than sporadic MPNST. Radiother Oncol.

[14] Yuan ZN, Xu LB, Zhao ZG, Xu SF, Zhang XX, Liu T, Zhang SG, Yu SJ (2017). Clinicopathological features and prognosis of malignant peripheral nerve sheath tumor: a retrospective study of 159 cases from 1999 to 2016. Oncotarget.

[15] Valentin T, Le Cesne A, Ray-Coquard I, Italiano A, Decanter G, Bompas E, Isambert N, Thariat J, Linassier C, Bertucci F (2016). Management and prognosis of malignant peripheral nerve sheath tumors: the experience of the French Sarcoma Group (GSF-GETO). Eur J Cancer.

[16] Fan Q, Yang J, Wang G (2014). Clinical and molecular prognostic predictors of malignant peripheral nerve sheath tumor. Clin Transl Oncol.

[17] Longhi A, Errani C, Magagnoli G, Alberghini M, Gambarotti M, Mercuri M, Ferrari S (2010). High grade malignant peripheral nerve sheath tumors: outcome of 62 patients with localized disease and review of the literature. J Chemother.

[18] Anghileri M, Miceli R, Fiore M, Mariani L, Ferrari A, Mussi C, Lozza L, Collini P, Olmi P, Casali PG (2006). Malignant peripheral nerve sheath tumors: prognostic factors and survival in a series of patients treated at a single institution. Cancer.

[19] Ramanathan RC, Thomas JM (1999). Malignant peripheral nerve sheath tumours associated with von Recklinghausen's neurofibromatosis. Eur J Surg Oncol.

[20] Grobmyer SR, Reith JD, Shahlaee A, Bush CH, Hochwald SN (2008). Malignant peripheral nerve sheath tumor: molecular pathogenesis and current management considerations. J Surg Oncol.

[21] Korfhage J, Lombard DB (2019). Malignant peripheral nerve sheath tumors: from epigenome to bedside. Mol Cancer Res.

[22] De Raedt T, Beert E, Pasmant E, Luscan A, Brems H, Ortonne N, Helin K, Hornick JL, Mautner V, Kehrer-Sawatzki H (2014). PRC2 loss amplifies Ras-driven transcription and confers sensitivity to BRD4-based therapies. Nature.

[23] Zhang M, Wang Y, Jones S, Sausen M, McMahon K, Sharma R, Wang Q, Belzberg AJ, Chaichana K, Gallia GL (2014). Somatic mutations of SUZ12 in malignant peripheral nerve sheath tumors. Nat Genet.

[24] Lee W, Teckie S, Wiesner T, Ran L, Prieto Granada CN, Lin M, Zhu S, Cao Z, Liang Y, Sboner A (2014). PRC2 is recurrently inactivated through EED or SUZ12 loss in malignant peripheral nerve sheath tumors. Nat Genet.

[25] Sohier P, Luscan A, Lloyd A, Ashelford K, Laurendeau I, Briand-Suleau A, Vidaud D, Ortonne N, Pasmant E, Upadhyaya M (2017). Confirmation of mutation landscape of NF1-associated malignant peripheral nerve sheath tumors. Genes Chromosom Cancer.

[26] Cleven AH, Sannaa GA, Briaire-de Bruijn I, Ingram DR, van de Rijn M, Rubin BP, de Vries MW, Watson KL, Torres KE, Wang WL (2016). Loss of H3K27 tri-methylation is a diagnostic marker for malignant peripheral nerve sheath tumors and an indicator for an inferior survival. Mod Pathol.

[27] Wang T, Yin H, Han S, Yang X, Wang J, Huang Q, Yan W, Zhou W, Xiao J (2015). Malignant peripheral nerve sheath tumor (MPNST) in the spine: a retrospective analysis of clinical and molecular prognostic factors. J Neuro-Oncol.

[28] Ma C, Ow A, Shan OH, Wu Y, Zhang C, Sun J, Ji T, Pingarron Martin L, Wang L (2014). Malignant peripheral nerve sheath tumours in the head and neck region: retrospective analysis of clinicopathological features and treatment outcomes. Int J Oral Maxillofac Surg.

[29] LaFemina J, Qin LX, Moraco NH, Antonescu CR, Fields RC, Crago AM, Brennan MF, Singer S (2013). Oncologic outcomes of sporadic, neurofibromatosis-associated, and radiation-induced malignant peripheral nerve sheath tumors. Ann Surg Oncol.

[30] Martin E, Coert JH, Flucke UE, Slooff WBM, Ho VKY, van der Graaf WT, van Dalen T, van de Sande MAJ, van Houdt WJ, Grünhagen DJ, Verhoef C (2020). A nationwide cohort study on treatment and survival in patients with malignant peripheral nerve sheath tumours. Eur J Cancer.

[31] Mowery A, Clayburgh D (2019). Malignant peripheral nerve sheath tumors: Analysis of the national cancer database. Oral Oncol.

[32] Yan PH, Huang RZ, Hu P, Liu FS, Zhu XL, Hu PZ, Yin HB, Zhang J, Meng T, Huang ZQ (2019). Nomograms for predicting the overall and cause-specific survival in patients with malignant peripheral nerve sheath tumor: a population-based study. J Neuro-Oncol.

[33] Stang A (2010). Critical evaluation of the Newcastle-Ottawa scale for the assessment of the quality of nonrandomized studies in meta-analyses. Eur J Epidemiol.

[34] Bergamaschi L, Bisogno G, Manzitti C, D'Angelo P, Milano GM, Scagnellato A, Cappelletti M, Chiaravalli S, Dall'Igna P, Alaggio R, et al. Salvage rates and prognostic factors after relapse in children and adolescents with malignant peripheral nerve sheath tumors. *Pediatr Blood Cancer*. 2018;65. 10.1002/pbc.26816 28926683

[35] Watson KL, Al Sannaa GA, Kivlin CM, Ingram DR, Landers SM, Roland CL, Cormier JN, Hunt KK, Feig BW, Guadagnolo BA (2017). Patterns of recurrence and survival in sporadic, neurofibromatosis Type 1-associated, and radiation-associated malignant peripheral nerve sheath tumors. J Neurosurg.

[36] Vasconcelos RAT, Coscarelli PG, Alvarenga RP, Acioly MA (2017). Malignant peripheral nerve sheath tumor with and without neurofibromatosis type 1. Arq Neuropsiquiatr.

[37] Hwang IK, Hahn SM, Kim HS, Kim SK, Kim HS, Shin KH, Suh CO, Lyu CJ, Han JW (2017). Outcomes of treatment for malignant peripheral nerve sheath tumors: different clinical features associated with neurofibromatosis type 1. Cancer Res Treat.

[38] Goertz O, Langer S, Uthoff D, Ring A, Stricker I, Tannapfel A, Steinau HU (2014). Diagnosis, treatment and survival of 65 patients with malignant peripheral nerve sheath tumors. Anticancer Res.

[39] Kamran SC, Howard SA, Shinagare AB, Krajewski KM, Jagannathan JP, Hornick JL, Ramaiya NH (2013). Malignant peripheral nerve sheath tumors: prognostic impact of rhabdomyoblastic differentiation (malignant triton tumors), neurofibromatosis 1 status and location. Eur J Surg Oncol.

[40] Stucky CC, Johnson KN, Gray RJ, Pockaj BA, Ocal IT, Rose PS, Wasif N (2012). Malignant peripheral nerve sheath tumors (MPNST): the Mayo Clinic experience. Ann Surg Oncol.

[41] Rekhi B, Ingle A, Kumar R, Desouza M, Dikshit R, Jambhekar NA (2010). Malignant peripheral nerve sheath tumors: clinicopathological profile of 63 cases diagnosed at a tertiary cancer referral center in Mumbai, India. Indian J Pathol Microbiol.

[42] Porter DE. Prasad V, Foster L, Dall GF, Birch R, Grimer RJ: Survival in malignant peripheral nerve sheath tumours: a comparison between sporadic and neurofibromatosis type 1-associated tumours. *Sarcoma*. 2009;2009. 10.1155/2009/756395 PMC2666272 19360115

[43] Keizman D, Issakov J, Meller I, Maimon N, Ish-Shalom M, Sher O, Merimsky O (2009). Expression and significance of EGFR in malignant peripheral nerve sheath tumor. J Neuro-Oncol.

[44] Okada K, Hasegawa T, Tajino T, Hotta T, Yanagisawa M, Osanai T, Nishida J, Seki K, Itoi E (2007). Clinical relevance of pathological grades of malignant peripheral nerve sheath tumor: a multi-institution TMTS study of 56 cases in Northern Japan. Ann Surg Oncol.

[45] Meis JM, Enzinger FM, Martz KL, Neal JA (1992). Malignant peripheral nerve sheath tumors (malignant schwannomas) in children. Am J Surg Pathol.

[46] Nambisan RN, Rao U, Moore R, Karakousis CP (1984). Malignant soft tissue tumors of nerve sheath origin. J Surg Oncol.

[47] Phillips B, Ball C, Sackett D, Badenoch D, Straus S, Haynes B, Dawes M. Oxford Centre for evidence-based medicine levels of evidence. *Revista Portuguesa De Clínica Geral*. 2001.

[48] Martin E, Coert JH, Flucke UE, Slooff WBM, van de Sande MA, van Noesel MM, Grunhagen DJ, Wijnen M, Verhoef C. Neurofibromatosis-associated malignant peripheral nerve sheath tumors in children have a worse prognosis: a nationwide cohort study. *Pediatr Blood Cancer*. 2019. 10.1002/pbc.28138 31889416

[49] Arshi A, Tajudeen BA, St John M (2015). Malignant peripheral nerve sheath tumors of the head and neck: demographics, clinicopathologic features, management, and treatment outcomes. Oral Oncol.

[50] Kar M, Deo SV, Shukla NK, Malik A, DattaGupta S, Mohanti BK, Thulkar S (2006). Malignant peripheral nerve sheath tumors (MPNST)--clinicopathological study and treatment outcome of twenty-four cases. World J Surg Oncol.

[51] Kolberg M, Holand M, Agesen TH, Brekke HR, Liestol K, Hall KS, Mertens F, Picci P, Smeland S, Lothe RA (2013). Survival meta-analyses for >1800 malignant peripheral nerve sheath tumor patients with and without neurofibromatosis type 1. Neuro-Oncology.

[52] Amirian ES, Goodman JC, New P, Scheurer ME (2014). Pediatric and adult malignant peripheral nerve sheath tumors: an analysis of data from the surveillance, epidemiology, and end results program. J Neuro-Oncol.

[53] Baehring JM, Betensky RA, Batchelor TT (2003). Malignant peripheral nerve sheath tumor: the clinical spectrum and outcome of treatment. Neurology.

