# Supplementary figures and images for: Prognosis and risk factors for malignant peripheral nerve sheath tumor: a systematic review and meta-analysis
Source: World J Surg Oncol. 2020 Sep 30;18:257. doi: 10.1186/s12957-020-02036-x (PMC7528472; doi:10.1186/s12957-020-02036-x)

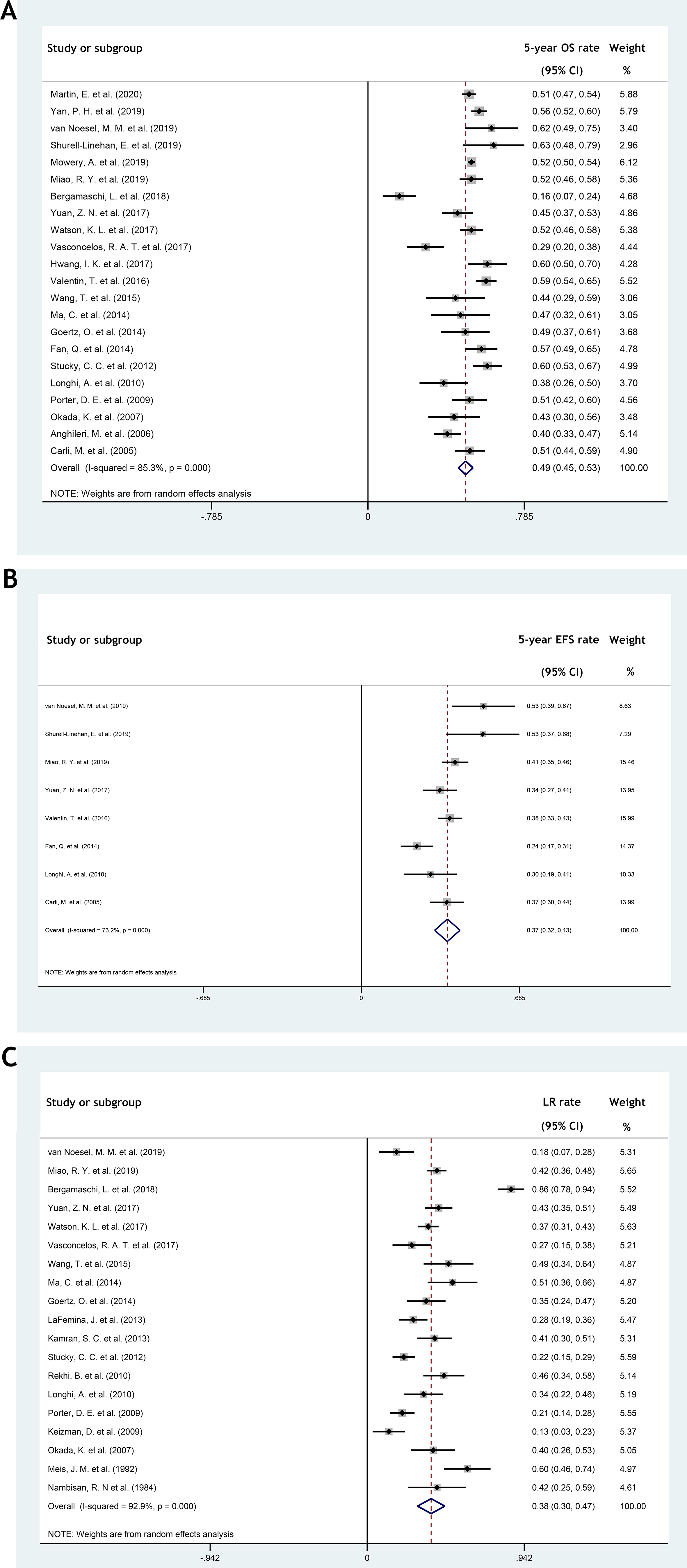

Supplement: Supplementary file 1 — Additional file 1: Figure S1. Forest plot showing the pooled rates: (A) 5-year OS rate. (B) 5-year EFS rate. (C) LR rate [file 12957_2020_2036_MOESM1_ESM.jpg]

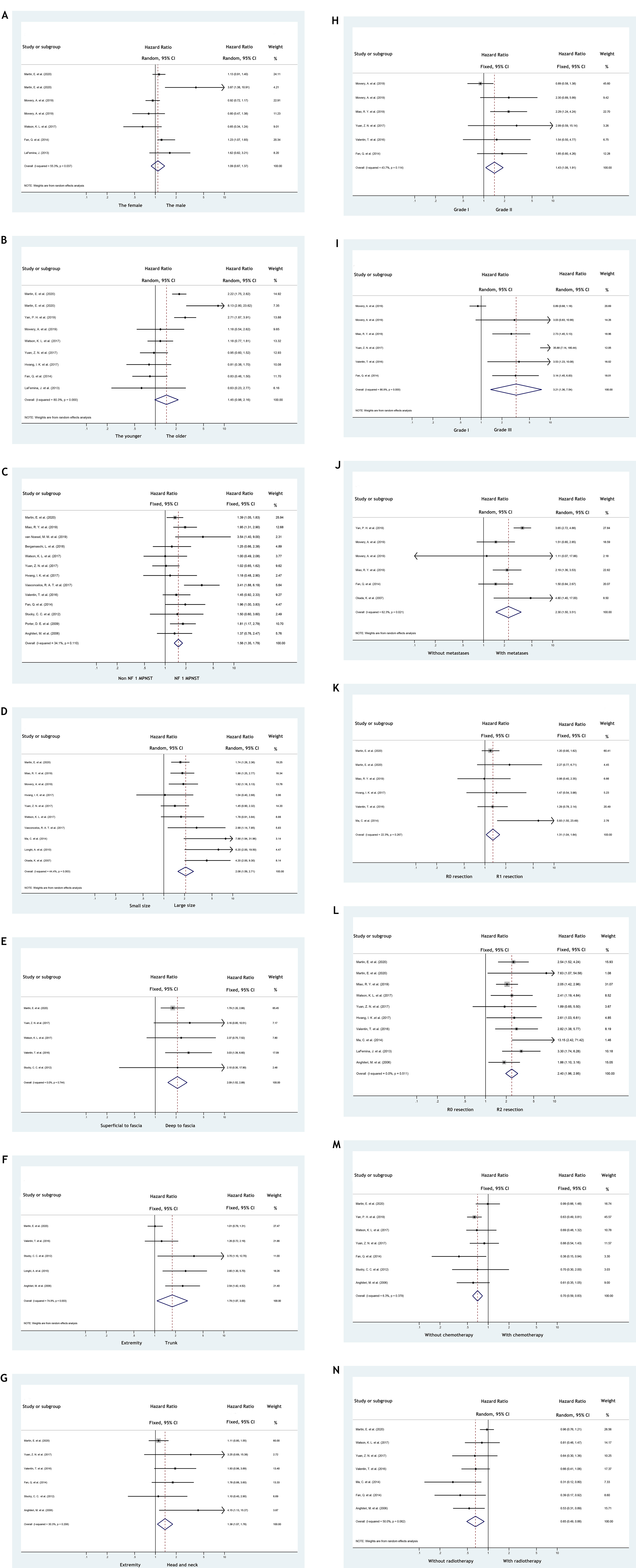

Supplement: Supplementary file 2 — Additional file 2: Figure S2. Forest plot showing the pooled HR of OS by prognostic factors: (A) Sex (The female vs. The male). (B) Age (The older vs. The younger). (C) NF 1 status (NF 1 vs. Non-NF 1 MPNST). (D) Tumor size (Large size vs. Small size). (E) Tumor depth (Deep vs. Superficial to fascia). (F) Tumor site (Trunk vs. Extremity). (G) (Head & neck vs. Extremity). (H) Tumor grade (Grade II vs. Grade I). (I) Tumor grade (Grade III vs. Grade I). (J) Metastases (With vs. Without). (K) Margin status (R1 vs. R0 resection). (L) Margin status (R2 vs. R0 resection). (M) Chemotherapy (With vs. Without). (N) Radiotherapy (With vs. Without) [file 12957_2020_2036_MOESM2_ESM.jpg]

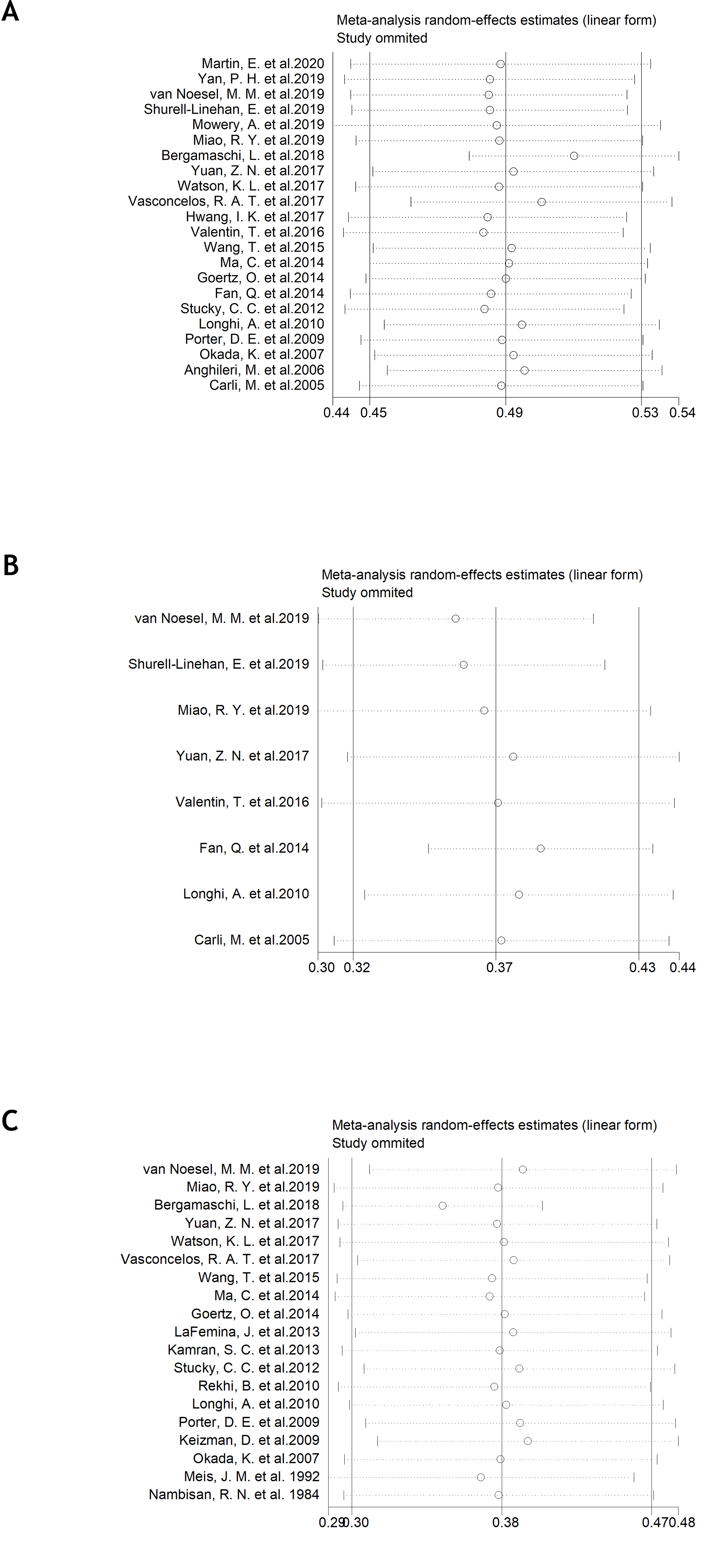

Supplement: Supplementary file 3 — Additional file 3: Figure S3. Forest plot for the sensitivity analysis in the meta-analysis: (A) 5-year OS rate. (B) 5-year EFS rate. (C) LR rate [file 12957_2020_2036_MOESM3_ESM.jpg]

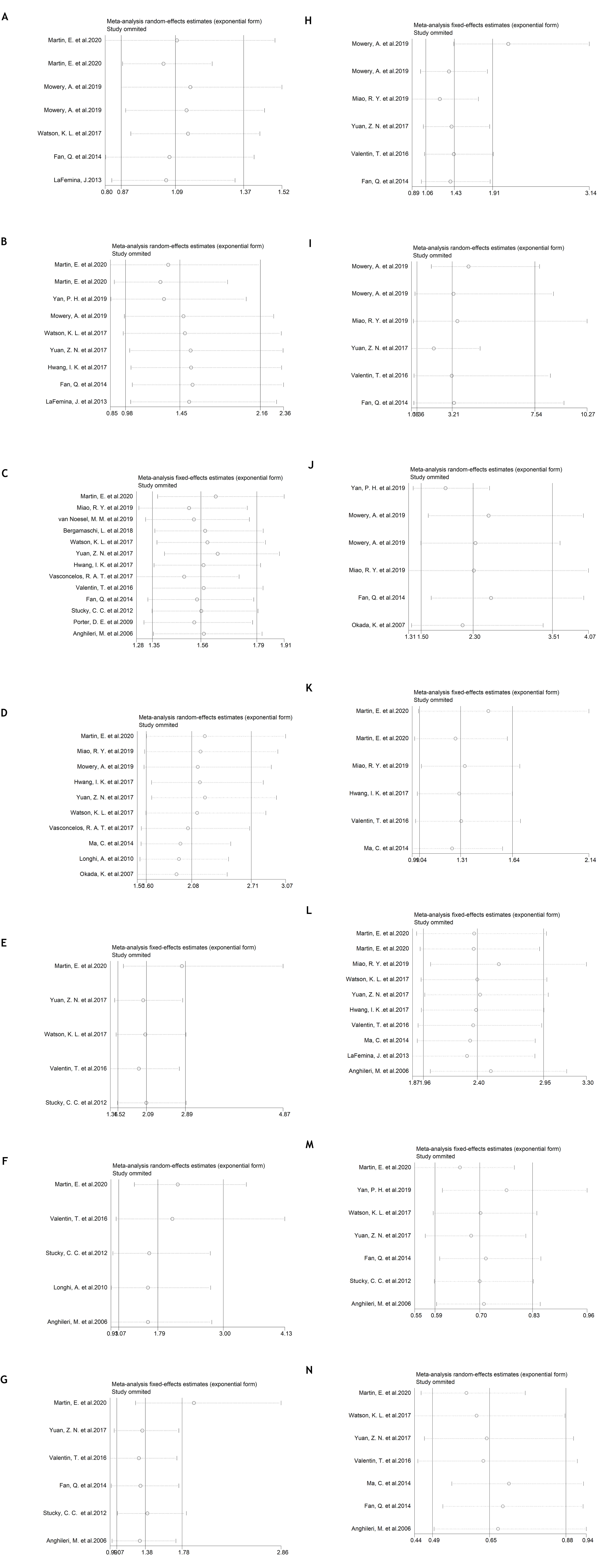

Supplement: Supplementary file 4 — Additional file 4: Figure S4. Forest plot for the sensitivity analysis in the meta-analysis: (A) Sex (The female vs. The male). (B) Age (The older vs. The younger). (C) NF 1 status (NF 1 vs. Non-NF 1 MPNST). (D) Tumor size (Large size vs. Small size). (E) Tumor depth (Deep vs. Superficial to fascia). (F) Tumor site (Trunk vs. Extremity). (G) (Head & neck vs. Extremity). (H) Tumor grade (Grade II vs. Grade I). (I) Tumor grade (Grade III vs. Grade I). (J) Metastases (With vs. Without). (K) Margin status (R1 vs. R0 resection). (L) Margin status (R2 vs. R0 resection). (M) Chemotherapy (With vs. Without). (N) Radiotherapy (With vs. Without) [file 12957_2020_2036_MOESM4_ESM.jpg]

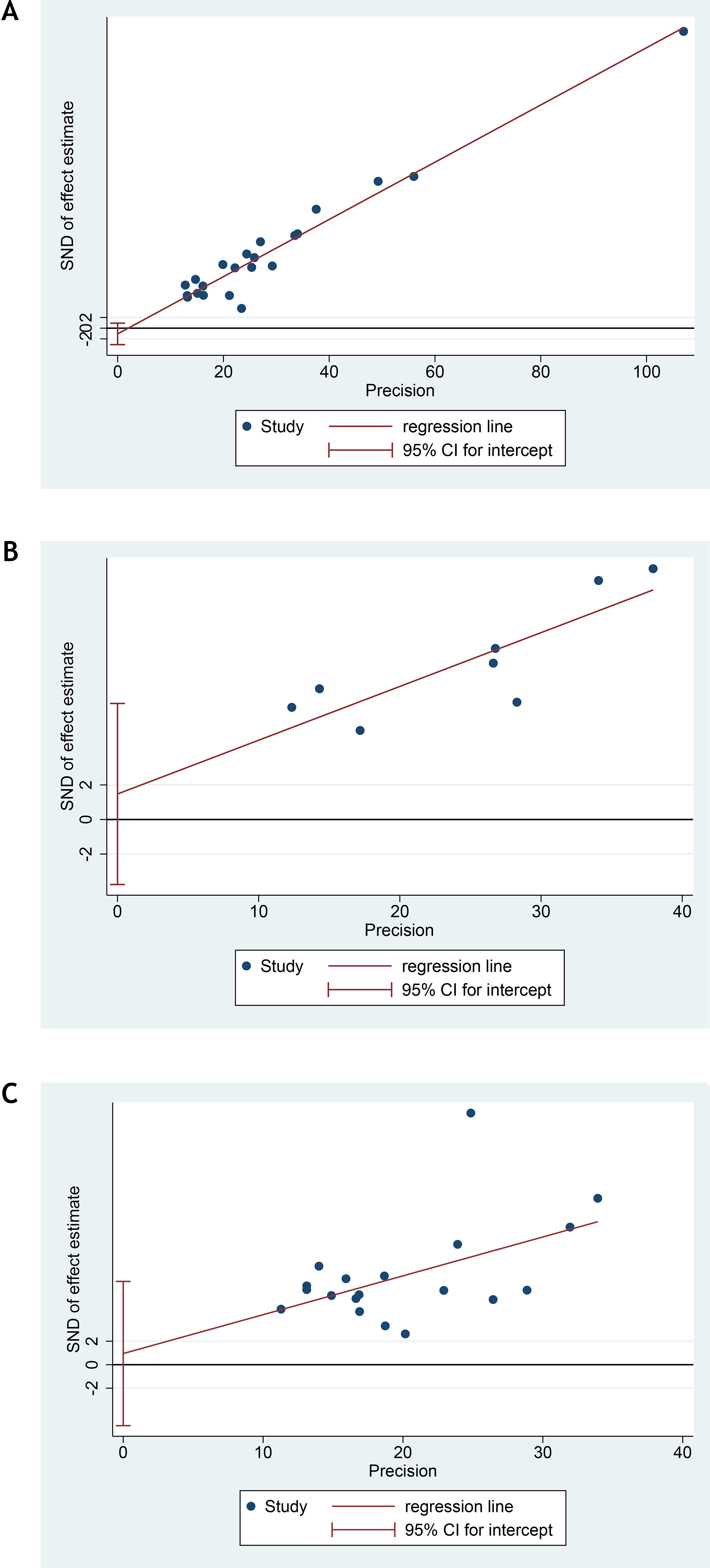

Supplement: Supplementary file 5 — Additional file 5: Figure S5. Egger’s test for publication bias in the meta-analysis: (a) 5-year OS rate. (b) 5-year EFS rate. (c) LR rate [file 12957_2020_2036_MOESM5_ESM.jpg]

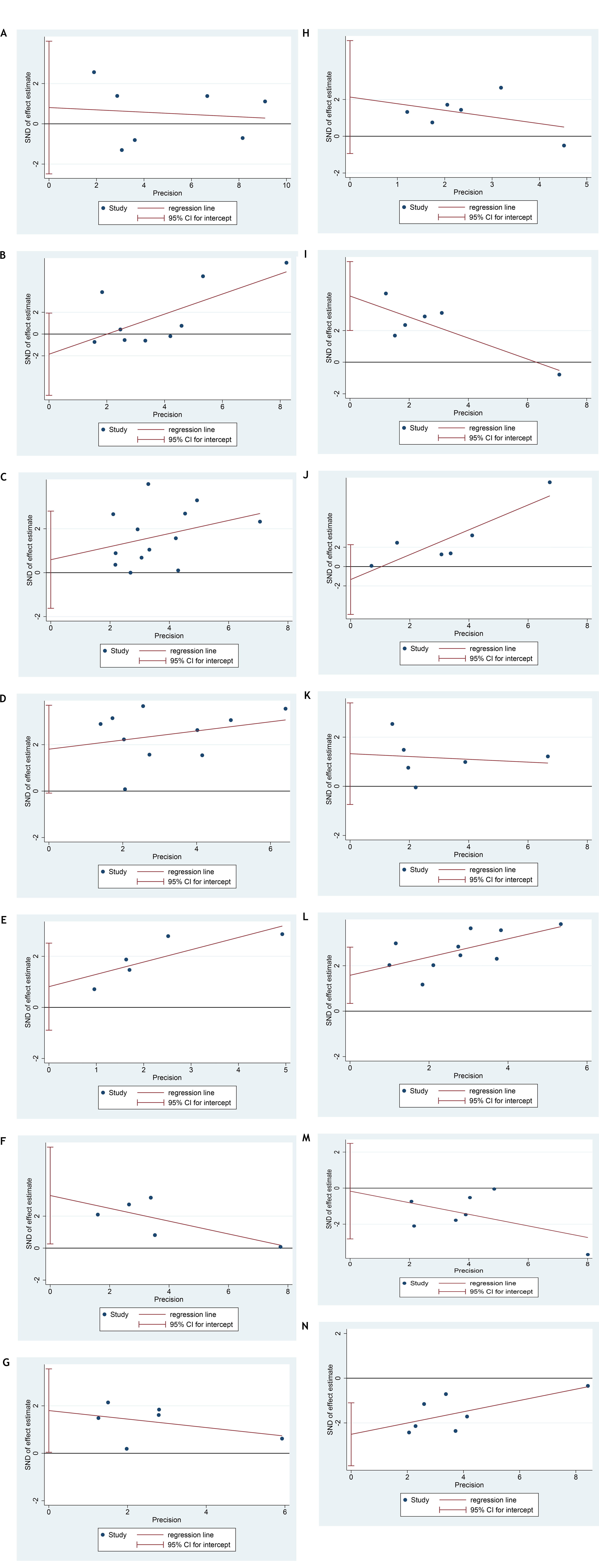

Supplement: Supplementary file 6 — Additional file 6: Figure S6. Egger’s test for publication bias in the meta-analysis: (A) Sex (The female vs. The male). (B) Age (The older vs. The younger). (C) NF 1 status (NF 1 vs. Non-NF 1 MPNST). (D) Tumor size (Large size vs. Small size). (E) Tumor depth (Deep vs. Superficial to fascia). (F) Tumor site (Trunk vs. Extremity). (G) (Head & neck vs. Extremity). (H) Tumor grade (Grade II vs. Grade I). (I) Tumor grade (Grade III vs. Grade I). (J) Metastases (With vs. Without). (K) Margin status (R1 vs. R0 resection). (L) Margin status (R2 vs. R0 resection). (M) Chemotherapy (With vs. Without). (N) Radiotherapy (With vs. Without) [file 12957_2020_2036_MOESM6_ESM.jpg]
